# Evolution of the risk of death and hospitalisation in drivers involved in road crashes in spain, 1993–2020: an age-period-cohort analysis

**DOI:** 10.1186/s40621-024-00552-y

**Published:** 2024-12-18

**Authors:** Luis Miguel Martín-delosReyes, Virginia Martínez-Ruiz, Mario Rivera-Izquierdo, Eladio Jiménez-Mejías, Nicolás Francisco Fernández Martínez, Pablo Lardelli-Claret

**Affiliations:** 1 Department of Preventive Medicine and Public Health, University of Granada, Granada, Spain; 2 Centros de Investigación Biomédica en Red de Epidemiología y Salud Pública (CIBERESP), Madrid, Spain; 3 Instituto de Investigación Biosanitaria de Granada (ibs.GRANADA), Granada, Spain; 4 Andalusian School of Public Health (EASP), Granada, Spain

**Keywords:** Road crashes, Age-period-cohort, Severity, Death, Hospitalisation

## Abstract

**Background:**

A prerequisite for understanding temporal changes in road crash severity is an unbiased description of this phenomenon. The aim of this study was to estimate the independent association trends of age, period and cohort with severity, encompassing the risk of death (RD) and the risk of death or hospitalisation (RDH) within 24 h, for drivers of passenger cars involved in road crashes with casualties in Spain from 1993 to 2020.

**Methods:**

The study population comprised 2,453,911 drivers of passenger cars aged 18 to 98 years involved in road crashes included in the registers of the General Directorate of Traffic. Crash- and driver-related variables with sufficient continuity over time were included, establishing RD and RDH as study outcomes. Temporal trends of both outcomes were analysed using multivariable Poisson regression and multivariable age-period-cohort intrinsic estimator models. An additional sensitivity analysis was performed for the subset of single crashes.

**Results:**

Severity estimates showed some variation across strategies. The APC model identified: (1) a J-shaped pattern for the effect of age on severity, (2) a decline in severity between 2001 and 2004 and 2013–2016, and (3) a birth cohort effect for both RD and RDH. In particular, the 1952–1958 cohort had the highest risk (RD = 1.17; 95%CI = 1.11–1.24 and RDH = 1.16; 95%CI = 1.13–1.19), followed by a decreasing trend in subsequent cohorts. Restricting the analysis to single crashes yielded similar results, with the exception of the age effect (severity increased with age). Furthermore, sex differences were observed–female sex was inversely associated with severity, especially for RD.

**Conclusions:**

RD and RDH decreased during the first decade of the 21st century, but seemed to stabilise from 2013 onwards. Evidence from this study support that birth cohort is associated with road crash severity, independent of age and period. This cohort effect might be due, at least partially, to improvements in general and road safety education. Further studies are needed to elucidate the causes of our findings and to identify factors accounting for sex differences.

**Supplementary Information:**

The online version contains supplementary material available at 10.1186/s40621-024-00552-y.

## Introduction

Spain is a South European country characterised by population ageing, with an old-age dependency ratio of 30.2% in 2022 [1]. In the same year, a total of 97,916 road crashes resulted in 1,746 fatalities, yielding a crude mortality rate of 37 per million inhabitants, below the European Union average [2]. The severity of road crashes in Spain has apparently decreased in recent decades. According to the latest Road Crashes Yearbook published by the General Directorate of Traffic (DGT, initials in Spanish), the number of fatalities per 100 road crash victims has fallen from 5.2 in 1993 to 1.3 in 2022 [3]. Although much of this decline is due to a progressive increase in the registration of minor injuries rather than a real decrease in the probability of death of those involved in crashes [3], there are many reasons that could justify actual changes in the severity of road crashes over time: changes in the demographic structure and other characteristics of the population involved, the speed at which crashes occur, driving styles, the active and passive safety features of vehicles and their use, or the quality of health care provided to victims [4–7]. To address the specific contribution of each one of these factors, a detailed and unbiased description of the evolution of road crash severity over time can first be made by applying an age-period-cohort (APC) approach.

Age-period-cohort analyses have been extensively used to study the temporal evolution of health indicators (mainly, mortality rates). Their aim is to identify the independent association that each of the three time dimensions involved (the time at which the indicator is estimated and the age and year of birth of the persons for whom it is measured) has with the magnitude of the indicator, on the assumption that different causal effects underlie each of these three possible associations. In the context of road crash morbidity and mortality, such analyses have been used to explain changes in mortality rates [5, 7, 8] or the incidence of deaths or injuries in different user groups with respect to the total population [9, 10] or the vehicle fleet [11], but we have not found any study that applies this methodology to an indicator of road crash severity (i.e. unaffected by either the intensity of exposure or the risk of being involved in a crash). If the probability of death or serious injury (requiring hospitalisation) are used as estimators of severity, it seems reasonable to assume that most of the causal effect of changes in vehicle- and environment-dependent factors (mainly road conditions, health care and legislation) over time should be captured by the period effect [4, 12, 13]. In addition, the possible association between age and injury severity would depend on two factors: the physical vulnerability (intrinsic or acquired) of the persons involved in the crash and, specifically for drivers, the association between age and driving style [14]. Both factors, but especially the latter, could also support the existence of a cohort effect.

Considering the above, the aim of this study is to estimate, by applying an APC analysis, the independent association trends of age, study period and birth cohort with severity, defined as the probability of death or hospitalisation within 24 h, for drivers of passenger cars involved in road crashes in Spain from 1993 to 2020. All crashes with casualties occurring in this period were sampled and adjusted for possible bias due to changes in the coverage of the register over time.

## Methods

### Study design and population

We have studied the case series consisting of the 2,453,911 drivers of passenger cars aged between 18 and 98 and of known sex involved in road crashes in which there were no pedestrians implicated, included in the registers of road crashes with victims produced annually by the DGT between 1993 and 2020 from the Statistical Bulletins that state police forces fill in at the scene of the crash. Throughout the study period there have been various changes that have affected both the information collected in the aforementioned bulletins and the system for coding, sending and storing this information in the register. The new system (ARENA2) improved data traceability and included automatization to remove duplicates and to reduce missing data in vehicle- and driver-related fields. However, it also introduced changes in the structure of data files and in the categorisation of several variables (e.g., nationality), none of which were included in this study. Moreover, these changes have occurred asynchronously in some Autonomous Communities. All of this has made it difficult to generate a single database for the entire study period that would be minimally affected by these changes. To overcome this drawback, we have been forced to select information from just a few sets of variables for which sufficient continuity over time was observed:

- Of the crash: identification code (unique identifier of each road crash, enabling linkage with the vehicles and persons involved), year (calendar year) and province (an administrative subdivision of Autonomous Communities), type of crash (single or multiple), number of vehicles involved and number of fatalities and serious injuries as a result of the crash.
- Of the drivers: injuries (categorised into uninjured/slightly injured if they did not require hospital admission, or seriously injured if they required hospital admission or died within 24 h of the crash), age in years and sex.

From the previous variables, the following new variables were constructed: the year of the crash was categorised into 4-year strata (1993–1996, 1997–2000, 2001–2004, 2005–2008, 2009–2012, 2013–2016, and 2017–2020). Similarly, age was categorised into 4-year age strata starting at age 18 [18 to 21, 22 to 25, etc.] except for the last stratum, which ranged from age 78 to 98. From the difference of the two previous variables, the birth cohort variable was generated, grouping individuals born in partially overlapping 7 calendar year strata (with 3 years common to each two contiguous strata), from those born between 1996 and 2002 to births between 1940 and 1956. For the birth cohorts prior to the latter, as the last age group comprises 78 to 98 years of age, the strata are wider and with a greater number of overlapping years: 1925–1942, 1916–1938, 1911–1934, 1908–1930, 1904–1926, 1900–1922, and 1900–1918. Finally, for each driver, the following variables were created: the number of fatalities, the number of seriously injured persons (i.e., requiring hospitalisation), and the sum of both amounts divided by the remaining persons involved in the same crash.

### Analysis

Taking as outcomes (dependent variables) the risk of death of the driver within 24 h (RD) and the risk of death or hospitalisation (RDH), two complementary analytic strategies were applied:

- A conventional Poisson regression model including as independent variables year, age groups, sex, the number of fatalities or serious injuries among the rest of individuals involved in the same crash (categorised as 0, 1, 2 or more than 2) and the province in which the crash occurred. This model assumes no effect of birth cohort on RD.
- An APC analysis. Many strategies have been proposed to solve the identification problem generated by the linear dependence of the three elements of analysis (birth cohort = period - age). We have chosen the APC intrinsic estimator (APC-IE) model, developed by Yang et al. [15], already used in previous studies to analyse road crash mortality rates [5, 7] and motorcyclist injuries [9]. Unlike classical APC analysis, the APC-IE model does not require a priori restrictions on the effect of any of the three time dimensions in order to circumvent identifying constraints. The APC-IE model is based on a principal component analysis, obtained after an orthogonal transformation of the original variables. The regression results of these components, which are no longer linearly correlated to one another, are then transformed to the original space by decomposing their eigenvalues [3]. From a Poisson model that includes age and age groups as predictors, Stata’s *apc_ie* command generates the corresponding cohort indicator variable and estimates the exponentiated coefficients (along with their 95% confidence interval, CI) for each age stratum, period and cohort. These coefficients are interpretable as relative risks (RRs): the ratio between the risk of the outcome in each stratum and the average risk for the strata as a whole. The province and the number of fatalities or serious injuries among all those involved in the same crash were included in all models to partially control bias introduced by the different coverage and quality of the information collected by the police across Spanish provinces, as well as by the inclusion over time in the register of crashes with no fatalities or serious injuries, or of people involved in crashes who were not deceased or seriously injured. Driver sex was also included in all models.

Finally, all the above models were built for single crashes only, where RD and RDH are not affected by the role of the other drivers involved in the same crash. All analyses were performed with the statistical software Stata (version 17) [16].

## Results

**Fig. 1:**
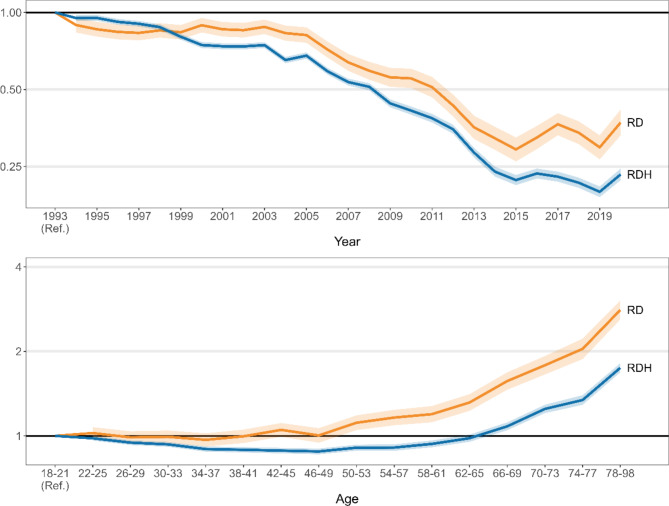
Relative risk of death (RD) and of death or hospitalisation (RDH) obtained from Poisson regression models. It shows the estimates obtained from multivariable Poisson regression models, adjusted for driver sex, number of fatalities or serious injuries divided by the remaining persons involved in the same crash, and province. The relative risk of each outcome is presented by year (top) and driver age (bottom)

Figure 1 (top) shows the RRs for each year since 1993 for RD and RDH, obtained using conventional Poisson regression models (an additional table file shows the values of all estimates used in Fig. 1, together with their 95% CI [see Additional file 1]). Taking RD as the outcome, RRs maintained fairly stable values between 1994 and 2003, declined sharply between 2003 and 2015, and showed an irregular pattern between 2015 and 2020. For RDH, RRs declined from 1993 to 2015, although more markedly from 2005 to 2015. For both outcomes, a remarkable increase in RR was observed in 2020 compared to the previous year. For the study period as a whole, RR reduction was 63% for RD and 77% for RDH.

Figure 1 (bottom) shows the RRs obtained with the same model for each age stratum from the 18–21 years. For RD, RRs remained around 1 up to the 46–49 age stratum and began to increase more or less exponentially thereafter. For RDH, a slight decrease in RRs was observed between the 18–21 and the 46–49 age strata. From this age group onwards, RRs increased exponentially with age, although less markedly than for RD.

**Fig. 2:**
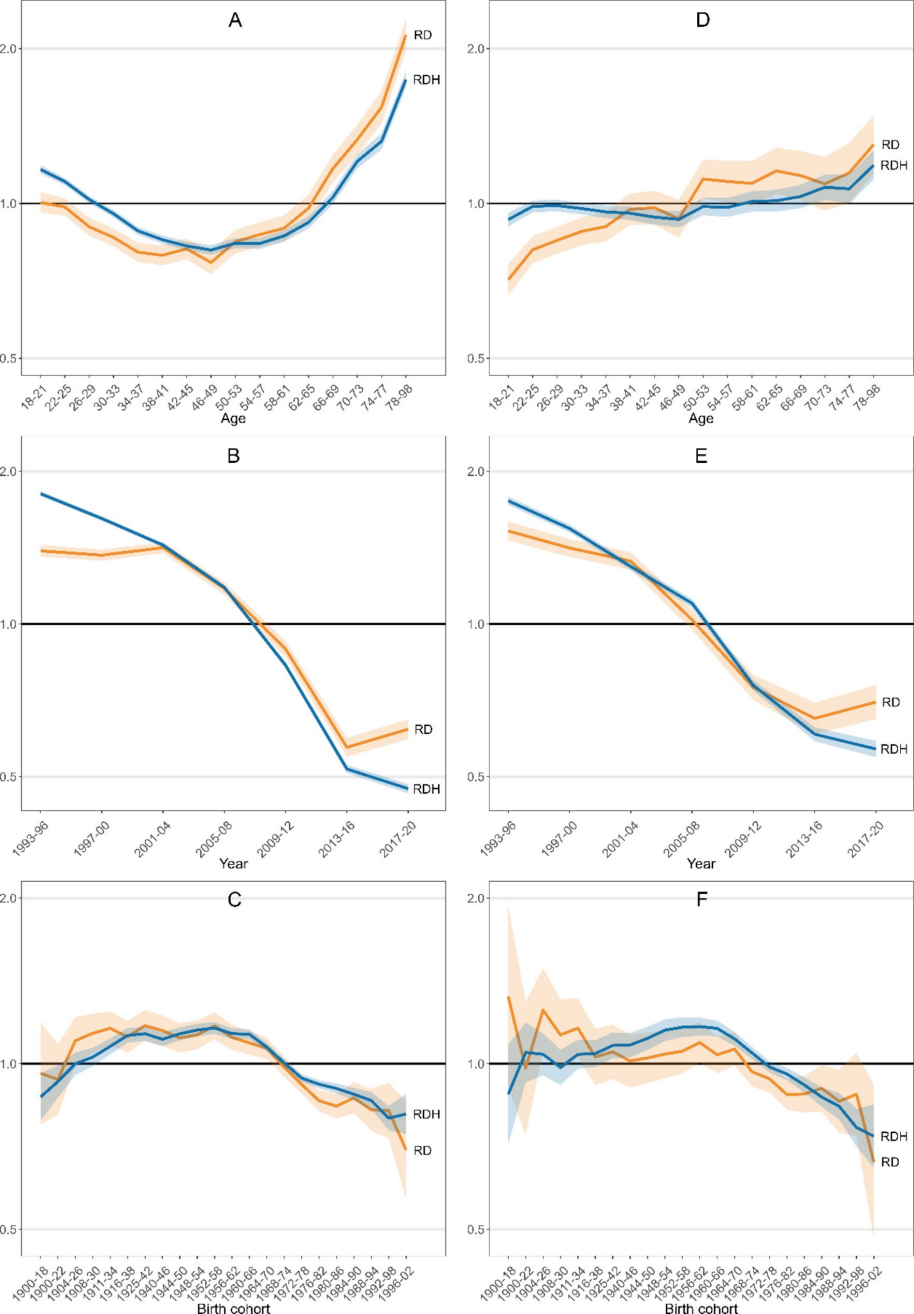
Relative risk of death (RD) and of death or hospitalisation (RDH) obtained from age-period-cohort models. It shows the estimates obtained from multivariable age-period-cohort intrinsic estimation models adjusted for driver sex, number of fatalities or serious injuries divided by the remaining persons involved in the same crash, and province. The relative risk of each outcome is presented by driver age (top), period (middle) and birth cohort (bottom). The left panel (**A**, **B**, **C**) shows results for all road crashes analysed in this study, while the right panel (**D**, **E**, **F**) shows only single crashes

Figure 2 (A, B and C) displays the results of the APC-IE models for the whole population of drivers, with the same adjustment variables as in the previous models (an additional table file shows the values of all estimates used in Fig. 2, along with their 95% CI [see Additional file 2]). Regarding age (Fig. 2A), a J-shaped pattern was observed for both RD and RDH: a decrease in RRs between the 18–21 and 46–49 age strata (more marked for RDH) and an increase from this age up to the 78–98 age group. An example is provided to clarify the interpretation of RRs: the 74–77 age stratum (RD = 1.54) had a 54% higher probability of dying than the average across strata, after accounting for the period and cohort effects. The period effect (Fig. 2B) showed a pattern consistent with that obtained from conventional Poisson regression models: for both RD and RDH, a marked decline in RRs was observed between the four-year periods 2001–04 and 2013–16. In the first two four-year periods (1993–96 and 1997–00), the decline was only observed for RDH. In the last four-year period, RDH decreased slightly compared to RD, which showed a slight increase. Finally, although at a lesser extent two previous two, a cohort effect was also observed (Fig. 2C): an increase in RDH was observed from the oldest cohort to the 1952–1958 one and a from the 1960–66 cohort onwards. The decline in RR from the 1952–58 to the 1996–02 cohort was also observed for RD, although the upward trend prior to 1952 was not as clear for this outcome.

When the analysis was restricted to drivers involved in single crashes (Fig. 2, D, E and F), RD increased with age (Fig. 2D), although irregularly. For RDH, after an initial increase between the first and second age strata, RRs decreased slightly up to 46–49 years of age and increased onwards. Regarding the period effect (Fig. 2E), a steady decline in RRs was observed for RDH over the entire period, only attenuated in the last four years. For RD, RRs also decreased slightly in the first two four-year periods but continued to increase in the last four-year period. Finally, the pattern of the cohort effect for RDH (Fig. 2F) was similar to that described for crashes as a whole. For RD, the pattern was more irregular, although the decline in RRs from the 1956–62 cohort onwards seemed to be evident.

For crashes as a whole, being a woman was inversely associated with RDH and especially with RD, with RRs of 0.87 (95% CI 0.86–0.88) and 0.57 (95% CI 0.55–0.59), respectively (the results were identical in the conventional Poisson model and in the APC model). These associations were even stronger for single crashes: 0.74 (95%CI 0.73–0.76) for RDH and 0.41 (95%CI 0.39–0.44) for RD.

## Discussion

### Main results

Our study has shown that, in addition to period, age and birth cohort are significantly associated with RD and RDH of drivers involved in road crashes with victims in Spain. These results are not directly comparable to other APC analyses conducted in other publications because that we have not been able to identify studies that use an indicator of crash severity not affected by either the intensity of exposure or the risk of being involved in a crash, although the results of studies using population road crash death rates as an outcome are quite similar to ours. For example, also applying an APC-IE model, Eun [5] observed a decline in population mortality rates in South Korea between 1988 and 2017, a J-shaped pattern for the age effect among 20–80 + year-olds, and a cohort effect with an inverted V-shaped pattern: increasing case fatality for successive cohorts from 1903 to 1943 and declining thereafter until the most recent cohort (2013). Using the same analytic strategy, in the United States, Macinko et al. [7] described a U-shaped pattern for driver fatality rates for 20–75 + year-olds and a decline for more recent birth cohorts (1955–2010), although they did not observe a decline in the period overlapping with our study (1990 to 2010). Using an outcome similar to ours (deaths and hospitalisations) but only for motorbike crashes, and taking the general population as the denominator, Langley et al. [9] also detected a declining cohort effect in New Zealand between 1959 and 1993.

The decrease in RD and RDH detected in our study is consistent with the reduction in road crash morbidity and mortality rates observed in other countries [17–19] and with previous research in Spain with different methodological approaches and period explored. For example, using a joinpoint regression model in census drivers, Barrio et al. [4] detected a decrease in driver fatality rates with respect to the total number of drivers registered in Spain between 2001 and 2011, with the trend accelerating in 2003 for alcohol-related deaths. These authors indicate that, probably, legislative changes in 2004 concerning the obligation to undergo breathalyser tests, the criminalisation of drunk driving and speeding, or the introduction of the demerit point system in 2006 may be partly responsible for this trend. It is important to note that, contrary to the hypotheses proposed in this and other studies to explain this decline, intensity of exposure (km or hours driven) cannot explain this trend in our study, as our at-risk population comprises drivers who have already been involved in a road crash. However, as indicated in the introduction, there are other factors that may explain this: improvements in infrastructure, better vehicles and health care, or greater use of safety measures in Spain [4, 20, 21] and other countries [5, 7, 19]. Finally, regarding the period effect, the downward trend slowed down or even reversed for mortality in the last four-year period (2017–2020). As explained in the limitations of the study, this phenomenon may be the result of a bias, as well as the distorting impact of the 2020 COVID-19 pandemic, making it necessary to explore other possible explanations.

The greater severity of injuries in older drivers is also consistent with previous studies for different types of crashes [6, 22–24]. The marked increase in RDH after the age of 50 may be due to increased frailty and lower tissue resistance to the impact of the energy involved, as well as to previous pathologies or increased drug use. Under the age of 50, the association showed in our analyses with age is variable depending on the method of analysis (Poisson regression vs. APC analysis), the outcome assessed (RD vs. RDH) or the type of crash (all road crashes vs. single crashes). Taking RDH as the outcome for all crashes, both the conventional and the APC model show a modest downward trend between the ages of 18 and 49, more pronounced in the latter model. Also in Spain, Santolino et al. [22] have found that drivers in the extreme age groups are associated with a higher risk of injury among the occupants of the vehicles they drive. In Switzerland, Spoerri et al. [6] found a U-shaped pattern in their road crash fatality rates. This pattern is consistent with the previous observation that road crashes involving younger drivers are inherently more severe than those involving older drivers [25]. This could be due to their driving at higher speeds or in environments where driving speeds are higher (e.g. more on roads than in urban areas) as well as to driving less safe vehicles. Related to this hypothesis, Farmer [26] observed that driver fatality rates are higher when driving smaller vehicles, which are more frequently used by younger drivers. When RD alone is considered as an outcome, a similar pattern to that described for RDH is observed in the APC analysis for all crashes. However, for single crashes, RD increased with age from 18 to 21 years onwards. It is possible that RD in single crashes, where injury severity does not depend on variables associated with other drivers involved in the same crash, better reflects the biological effect of ageing on injury severity as shown by other studies focused on single crashes [27–29].

Perhaps the major novel finding of our study is the identification of a cohort effect, independent of period and driver age, although, as expected, the range of variability in RR estimates associated with this effect is smaller than that observed for the other two dimensions. For both RD and RDH, cohorts of drivers born after the 1952–58 cohort have increasingly lower values. This pattern, consistent with the cohort effect detected in previous studies for other indicators related to the severity of road crash injuries [5, 7, 9], may have several explanations and further studies using additional sources of information are required in order to verify which factors related to both crash severity and birth cohort lies on the ground of the cohort effect detected in our study (i.e., different use of seat belts, different types of collisions or different driving environments, among others). However, given that our at-risk population consists of drivers already involved in road crashes, it is plausible to think of a progressively lower vulnerability to the energy effect as the date of birth becomes more recent, linked to an improvement in nutritional patterns. It is also plausible that improved education, both in general and in road safety, has led to a greater perception of the risks associated with driving in more recent birth cohorts. Previous studies [6, 30, 31] have shown an association between lower educational attainment and higher fatality rates, partly attributable to a higher frequency of engaging in driving styles associated with higher crash severity (e.g., driving without a seatbelt or under the influence of alcohol). Improvements since the decade of the 1950s in both factors have been documented in the Spanish context [32, 33].

Finally, our study shows lower injury severity when the driver was female, especially when the outcome assessed is RD. This result is also consistent with that described in studies that have used APC models for road crash fatality rates [5, 7], as well as others using different methodologies [6, 23, 25, 34, 35]. This may be due to less aggressive driving by women compared to men [36], as well as to the fact that women drive more frequently in environments where crashes tend to be less severe (e.g. in urban areas or during the day) [34, 37].

### Strengths and limitations

One of the main strengths of this study is the use of the APC-IE model, which does not require strong a priori information (and is thus preferred for exploratory analysis), yields unbiased estimates of finite-time-period APC analyses, has a smaller variance than estimators from any conventional generalised linear models and is asymptotically consistent [15]. Besides, it was applied to individual records (people involved in road crashes), rather than aggregate data. Additional strengths include the study’s nationwide character and long period coverage. Moreover, since the outcomes were conditional on having suffered a crash, we can limit the range of explanatory hypotheses for the APC effects to those not based on differences in exposure or risk of suffering a crash, often adduced in studies that use population mortality rates as the outcome [5, 8].

The most relevant limitations of our study relate to the source of information. For the whole period considered, the register used only has information on fatalities within 24 h after the crashes and not at 30 days. Furthermore, the quality of the register is heterogeneous in terms of the number and type of fatal crashes and the number and type of persons involved in the crashes. These data vary across provinces and depending on the calendar year (due to changes in the recording system). While the information on the number of deaths or hospitalisations seems comparable from year to year, the same is not true for the population at risk (people involved in casualty road crashes). It should be noted that a new data recording and reporting system (ARENA2) was implemented from 2014 onwards, although in some Autonomous Communities, this system coexisted with the previous one in 2014 and 2015. This results in artificial temporal variability that may be introduced in the data obtained before and after its implementation, aggravated by the circumstance that in some Autonomous Communities it was implemented later than in others. In addition, the information coverage is probably associated with the severity of the crash (i.e., more serious crashes have a higher probability of being recorded). All this may have led to a selection bias, although we cannot rule out, in addition, a possible information bias. To minimise the impact of these biases, we limited our analysis to drivers of passenger cars and excluded non-driving passengers and drivers of other types of vehicles, which showed greater variability in their registration from one year to the next. In addition, all models were adjusted for the province of origin of the data, as well as for the overall severity of the crash (measured by the number of fatalities and hospitalisations divided by the remaining persons involved in the same crash). Another limitation of this study is that the APC-IE model has some drawbacks, as it is hard to test whether its assumptions are verified in theory-driven scenarios and the results produced take the point closest to zero for each of the three time dimensions [38].

## Conclusions

The results of this study indicate a decrease in RD and RDH during the first decade of the 21st century, which could reflect the effectiveness of legislative changes aimed to minimise the impact of road crash injuries in Spain. However, the apparent stabilisation in the reduction of fatalities and hospitalisation since 2013 suggests the need to explore new strategies and reinforce existing efforts implementation and increase investment in road safety, vehicle and health care improvements. Moreover, in a progressively ageing driver population, the greater severity found among the oldest drivers may justify the development of vehicle safety features adapted to the elderly. On the other hand, the higher RD and RDH of the youngest drivers requires and in-depth analysis and better control of factors such as inexperience, speeding, driving in higher risk environments or in less safe vehicles. Furthermore, if improved general and road safety education is responsible for the cohort effect detected, this might provide an argument in favour of implementing road safety education as a basic cross-cutting element of the Spanish education system at an early age. Nevertheless, further studies are required to confirm this hypothesis and better understand the underlying factors. Finally, differences in RD and RDH between men and women merit further investigation to identify associated factors: driving styles, environments, vehicles, use and effectiveness of restraint systems, or intrinsic resistance to impact, among others.

## Electronic supplementary material

Supplementary Material 1

Supplementary Material 2

## Data Availability

The data belongs to the Spanish General Directorate of Traffic (DGT). DGT have not granted the authors permission to share the data.

## Abbreviations

APC: Age-period-cohort
CI: Confidence interval
DGT: General Directorate of Traffic
IE: Intrinsic estimator
RD: Risk of death
RDH: Risk of death or hospitalisation

## Declarations

This study does not contain data (i.e., details, images or videos) from any individual person.
