# Supplementary material for: Evolution of the risk of death and hospitalisation in drivers involved in road crashes in spain, 1993–2020: an age-period-cohort analysis
Source: Inj Epidemiol. 2024 Dec 18;11:67. doi: 10.1186/s40621-024-00552-y (PMC11653979; doi:10.1186/s40621-024-00552-y)
Supplement: Supplementary file 1 — Supplementary Material 1 [file 40621_2024_552_MOESM1_ESM.docx]

**Additional files of the manuscript titled ‘*Evolution of the risk of death and hospitalisation in drivers involved in road crashes in Spain, 1993–2020: an age-period-cohort analysis’*.**

Additional file 1. Total drivers, deaths within 24 hours and hospitalisations by year, age group and birth cohort. Results from conventional Poisson regression models.

| Year | Drivers, N | Deaths, N | Hospitalisations, N | RD | 95%CI | | RDH | 95%CI | |
| --- | --- | --- | --- | --- | --- | --- | --- | --- | --- |
|  |  |  |  |  |  | |  |  | |
| 1993 | 72651 | 1877 | 11692 | 1 |  |  | 1 |  |  |
| 1994 | 70461 | 1563 | 10494 | 0.89 | 0.84 | 0.96 | 0.95 | 0.93 | 0.98 |
| 1995 | 76481 | 1622 | 11373 | 0.86 | 0.80 | 0.92 | 0.95 | 0.93 | 0.98 |
| 1996 | 79552 | 1565 | 10994 | 0.84 | 0.78 | 0.90 | 0.92 | 0.90 | 0.94 |
| 1997 | 80195 | 1578 | 10947 | 0.83 | 0.78 | 0.89 | 0.90 | 0.88 | 0.93 |
| 1998 | 92951 | 1782 | 11895 | 0.85 | 0.80 | 0.91 | 0.88 | 0.85 | 0.90 |
| 1999 | 93819 | 1733 | 10854 | 0.84 | 0.78 | 0.89 | 0.80 | 0.78 | 0.82 |
| 2000 | 91672 | 1736 | 9565 | 0.89 | 0.83 | 0.95 | 0.75 | 0.73 | 0.77 |
| 2001 | 98871 | 1738 | 9862 | 0.86 | 0.80 | 0.92 | 0.74 | 0.72 | 0.76 |
| 2002 | 96902 | 1693 | 9675 | 0.85 | 0.80 | 0.91 | 0.74 | 0.72 | 0.76 |
| 2003 | 100918 | 1795 | 10137 | 0.88 | 0.82 | 0.94 | 0.75 | 0.73 | 0.77 |
| 2004 | 92612 | 1502 | 7910 | 0.83 | 0.78 | 0.89 | 0.65 | 0.63 | 0.67 |
| 2005 | 89179 | 1383 | 7791 | 0.82 | 0.76 | 0.87 | 0.68 | 0.66 | 0.70 |
| 2006 | 94695 | 1223 | 6918 | 0.72 | 0.67 | 0.77 | 0.59 | 0.57 | 0.61 |
| 2007 | 94654 | 1051 | 6118 | 0.64 | 0.59 | 0.69 | 0.53 | 0.52 | 0.55 |
| 2008 | 88734 | 872 | 5284 | 0.59 | 0.55 | 0.64 | 0.51 | 0.50 | 0.53 |
| 2009 | 85304 | 747 | 4179 | 0.56 | 0.51 | 0.61 | 0.44 | 0.43 | 0.46 |
| 2010 | 83745 | 703 | 3746 | 0.55 | 0.51 | 0.60 | 0.41 | 0.40 | 0.43 |
| 2011 | 79335 | 607 | 3282 | 0.51 | 0.47 | 0.56 | 0.39 | 0.37 | 0.40 |
| 2012 | 80873 | 510 | 2979 | 0.43 | 0.39 | 0.48 | 0.35 | 0.34 | 0.36 |
| 2013 | 86567 | 438 | 2548 | 0.36 | 0.32 | 0.40 | 0.28 | 0.27 | 0.30 |
| 2014 | 85111 | 444 | 2303 | 0.32 | 0.29 | 0.36 | 0.24 | 0.23 | 0.25 |
| 2015 | 89872 | 406 | 2189 | 0.29 | 0.26 | 0.32 | 0.22 | 0.21 | 0.23 |
| 2016 | 96826 | 443 | 2349 | 0.32 | 0.29 | 0.36 | 0.23 | 0.22 | 0.25 |
| 2017 | 95801 | 498 | 2267 | 0.37 | 0.33 | 0.40 | 0.23 | 0.22 | 0.24 |
| 2018 | 96907 | 460 | 2150 | 0.34 | 0.31 | 0.38 | 0.22 | 0.21 | 0.23 |
| 2019 | 95933 | 400 | 1966 | 0.30 | 0.27 | 0.33 | 0.20 | 0.19 | 0.21 |
| 2020 | 63290 | 343 | 1567 | 0.37 | 0.33 | 0.42 | 0.23 | 0.22 | 0.25 |
|  |  |  |  |  |  |  |  |  |  |
|  |  |  |  |  |  |  |  |  |  |
| Age Group |  |  |  |  |  |  |  |  |  |
| 18-21 | 204929 | 2782 | 18749 | 1 |  |  | 1 |  |  |
| 22-25 | 303105 | 3862 | 25719 | 1.02 | 0.97 | 1.07 | 0.98 | 0.96 | 1.00 |
| 26-29 | 294018 | 3406 | 22794 | 0.99 | 0.94 | 1.04 | 0.95 | 0.93 | 0.96 |
| 30-33 | 265756 | 2947 | 19343 | 0.99 | 0.94 | 1.05 | 0.93 | 0.91 | 0.95 |
| 34-37 | 241071 | 2499 | 16080 | 0.97 | 0.92 | 1.02 | 0.90 | 0.88 | 0.92 |
| 38-41 | 218523 | 2299 | 14130 | 1.00 | 0.94 | 1.05 | 0.89 | 0.87 | 0.91 |
| 42-45 | 192240 | 2140 | 12290 | 1.05 | 0.99 | 1.11 | 0.89 | 0.87 | 0.91 |
| 46-49 | 164440 | 1758 | 10436 | 1.00 | 0.95 | 1.07 | 0.88 | 0.86 | 0.90 |
| 50-53 | 140990 | 1699 | 9320 | 1.11 | 1.05 | 1.18 | 0.91 | 0.89 | 0.93 |
| 54-57 | 113805 | 1460 | 7559 | 1.16 | 1.09 | 1.24 | 0.91 | 0.88 | 0.93 |
| 58-61 | 92044 | 1264 | 6514 | 1.19 | 1.12 | 1.28 | 0.94 | 0.91 | 0.96 |
| 62-65 | 72322 | 1158 | 5640 | 1.32 | 1.23 | 1.41 | 0.98 | 0.95 | 1.01 |
| 66-69 | 52724 | 1035 | 4562 | 1.57 | 1.46 | 1.68 | 1.08 | 1.05 | 1.12 |
| 70-73 | 40559 | 889 | 3937 | 1.78 | 1.65 | 1.93 | 1.25 | 1.21 | 1.29 |
| 74-77 | 28343 | 684 | 2792 | 2.04 | 1.88 | 2.22 | 1.34 | 1.29 | 1.40 |
| 78-98 | 29042 | 830 | 3169 | 2.81 | 2.60 | 3.04 | 1.75 | 1.68 | 1.82 |
|  |  |  |  |  |  |  |  |  |  |
|  |  |  |  |  |  |  |  |  |  |
| Birth Cohort |  |  |  |  |  |  |  |  |  |
| 1900-18 | 1395 | 68 | 317 |  |  |  |  |  |  |
| 1900-22 | 3847 | 156 | 762 |  |  |  |  |  |  |
| 1904-26 | 9484 | 397 | 1718 |  |  |  |  |  |  |
| 1908-30 | 17457 | 647 | 2759 |  |  |  |  |  |  |
| 1911-34 | 31212 | 955 | 4217 |  |  |  |  |  |  |
| 1916-38 | 45977 | 1093 | 5362 |  |  |  |  |  |  |
| 1925-42 | 56976 | 1187 | 5642 |  |  |  |  |  |  |
| 1940-46 | 74461 | 1327 | 6689 |  |  |  |  |  |  |
| 1944-50 | 94905 | 1483 | 8213 |  |  |  |  |  |  |
| 1948-54 | 111023 | 1653 | 9382 |  |  |  |  |  |  |
| 1952-58 | 130388 | 1886 | 10830 |  |  |  |  |  |  |
| 1956-62 | 161642 | 2131 | 12975 |  |  |  |  |  |  |
| 1960-66 | 191433 | 2430 | 15571 |  |  |  |  |  |  |
| 1964-70 | 227608 | 2878 | 18398 |  |  |  |  |  |  |
| 1968-74 | 267732 | 3255 | 21426 |  |  |  |  |  |  |
| 1972-78 | 287608 | 3321 | 21844 |  |  |  |  |  |  |
| 1976-82 | 252309 | 2503 | 16450 |  |  |  |  |  |  |
| 1980-86 | 187478 | 1599 | 10190 |  |  |  |  |  |  |
| 1984-90 | 134830 | 946 | 5695 |  |  |  |  |  |  |
| 1988-94 | 90396 | 463 | 2767 |  |  |  |  |  |  |
| 1992-98 | 53317 | 242 | 1281 |  |  |  |  |  |  |
| 1996-02 | 22433 | 92 | 546 |  |  |  |  |  |  |
| Relative risks obtained from Poisson regression models adjusted for driver sex, province and number of fatalities or serious injuries divided by the remaining persons involved in the same crash (0, 1, 2, 3 or more).  RD: Risk of death within 24 hours. RDH: Risk of death or hospitalisation. | | | | | | | | | |
