# Supplementary material for: Evolution of the risk of death and hospitalisation in drivers involved in road crashes in spain, 1993–2020: an age-period-cohort analysis
Source: Inj Epidemiol. 2024 Dec 18;11:67. doi: 10.1186/s40621-024-00552-y (PMC11653979; doi:10.1186/s40621-024-00552-y)
Supplement: Supplementary file 2 — Supplementary Material 2 [file 40621_2024_552_MOESM2_ESM.docx]

**Additional files of the manuscript titled ‘*Evolution of the risk of death and hospitalisation in drivers involved in road crashes in Spain, 1993–2020: an age-period-cohort analysis’*.**

Additional file 2. Results of the APC-IE analysis for all crashes and for single crashes.

|  | All crashes | | | | | | Single crashes | | | | | |
| --- | --- | --- | --- | --- | --- | --- | --- | --- | --- | --- | --- | --- |
|  |  | | | | | |  | | | | | |
|  |  | | |  |  |  |  | | |  |  |  |
| Age | RD | 95%CI | | RDH | 95%CI | | RD | 95%CI | | RDH | 95%CI | |
|  |  |  | |  |  | |  |  | |  |  | |
| 18-21 | 1.00 | 0.96 | 1.05 | 1.16 | 1.14 | 1.19 | 0.71 | 0.66 | 0.76 | 0.93 | 0.90 | 0.96 |
| 22-25 | 0.98 | 0.95 | 1.02 | 1.10 | 1.09 | 1.12 | 0.81 | 0.77 | 0.86 | 0.99 | 0.96 | 1.01 |
| 26-29 | 0.90 | 0.87 | 0.94 | 1.02 | 1.00 | 1.03 | 0.85 | 0.80 | 0.90 | 0.99 | 0.97 | 1.02 |
| 30-33 | 0.86 | 0.83 | 0.89 | 0.96 | 0.94 | 0.97 | 0.88 | 0.83 | 0.94 | 0.98 | 0.95 | 1.00 |
| 34-37 | 0.80 | 0.77 | 0.84 | 0.88 | 0.87 | 0.90 | 0.90 | 0.84 | 0.96 | 0.96 | 0.94 | 0.99 |
| 38-41 | 0.79 | 0.76 | 0.83 | 0.85 | 0.83 | 0.87 | 0.97 | 0.91 | 1.04 | 0.96 | 0.93 | 0.99 |
| 42-45 | 0.82 | 0.78 | 0.86 | 0.83 | 0.81 | 0.84 | 0.98 | 0.91 | 1.06 | 0.94 | 0.91 | 0.97 |
| 46-49 | 0.77 | 0.73 | 0.81 | 0.81 | 0.79 | 0.83 | 0.93 | 0.86 | 1.02 | 0.93 | 0.90 | 0.97 |
| 50-53 | 0.84 | 0.80 | 0.89 | 0.84 | 0.82 | 0.86 | 1.12 | 1.02 | 1.22 | 0.99 | 0.95 | 1.03 |
| 54-57 | 0.87 | 0.82 | 0.92 | 0.84 | 0.82 | 0.86 | 1.10 | 1.00 | 1.21 | 0.98 | 0.94 | 1.03 |
| 58-61 | 0.90 | 0.84 | 0.95 | 0.86 | 0.84 | 0.89 | 1.09 | 0.99 | 1.21 | 1.01 | 0.96 | 1.06 |
| 62-65 | 0.98 | 0.92 | 1.04 | 0.92 | 0.89 | 0.94 | 1.16 | 1.04 | 1.28 | 1.01 | 0.96 | 1.06 |
| 66-69 | 1.17 | 1.10 | 1.24 | 1.03 | 1.00 | 1.06 | 1.13 | 1.01 | 1.26 | 1.03 | 0.98 | 1.09 |
| 70-73 | 1.33 | 1.24 | 1.42 | 1.21 | 1.17 | 1.25 | 1.09 | 0.97 | 1.23 | 1.08 | 1.02 | 1.14 |
| 74-77 | 1.54 | 1.43 | 1.66 | 1.32 | 1.27 | 1.37 | 1.15 | 1.00 | 1.31 | 1.07 | 1.00 | 1.14 |
| 78-98 | 2.13 | 1.98 | 2.28 | 1.74 | 1.68 | 1.80 | 1.30 | 1.14 | 1.48 | 1.19 | 1.11 | 1.27 |
|  |  |  |  |  |  |  |  |  |  |  |  |  |
|  |  |  |  |  |  |  |  |  |  |  |  |  |
| Period |  |  |  |  |  |  |  |  |  |  |  |  |
| 1993-96 | 1.39 | 1.36 | 1.43 | 1.81 | 1.79 | 1.83 | 1.53 | 1.46 | 1.59 | 1.75 | 1.72 | 1.78 |
| 1997-00 | 1.37 | 1.33 | 1.40 | 1.61 | 1.60 | 1.63 | 1.41 | 1.36 | 1.47 | 1.54 | 1.52 | 1.57 |
| 2001-04 | 1.41 | 1.38 | 1.45 | 1.43 | 1.41 | 1.45 | 1.33 | 1.28 | 1.38 | 1.30 | 1.28 | 1.32 |
| 2005-08 | 1.17 | 1.14 | 1.21 | 1.18 | 1.17 | 1.19 | 1.02 | 0.97 | 1.07 | 1.10 | 1.08 | 1.12 |
| 2009-12 | 0.89 | 0.86 | 0.92 | 0.83 | 0.82 | 0.84 | 0.75 | 0.71 | 0.79 | 0.76 | 0.74 | 0.78 |
| 2013-16 | 0.57 | 0.55 | 0.60 | 0.52 | 0.51 | 0.53 | 0.65 | 0.61 | 0.70 | 0.61 | 0.59 | 0.63 |
| 2017-20 | 0.62 | 0.59 | 0.65 | 0.47 | 0.46 | 0.48 | 0.70 | 0.65 | 0.76 | 0.57 | 0.55 | 0.59 |
|  |  |  |  |  |  |  |  |  |  |  |  |  |
|  |  |  |  |  |  |  |  |  |  |  |  |  |
| Birth Cohort |  |  |  |  |  |  |  |  |  |  |  |  |
| 1900-18 | 0.96 | 0.78 | 1.19 | 0.87 | 0.79 | 0.96 | 1.33 | 0.91 | 1.94 | 0.88 | 0.71 | 1.08 |
| 1900-22 | 0.94 | 0.81 | 1.08 | 0.93 | 0.87 | 0.99 | 0.98 | 0.74 | 1.30 | 1.05 | 0.92 | 1.19 |
| 1904-26 | 1.10 | 1.00 | 1.22 | 1.00 | 0.95 | 1.05 | 1.25 | 1.05 | 1.49 | 1.04 | 0.95 | 1.14 |
| 1908-30 | 1.14 | 1.05 | 1.23 | 1.03 | 0.99 | 1.07 | 1.13 | 0.97 | 1.31 | 0.98 | 0.91 | 1.06 |
| 1911-34 | 1.16 | 1.08 | 1.24 | 1.08 | 1.04 | 1.11 | 1.16 | 1.03 | 1.31 | 1.04 | 0.98 | 1.11 |
| 1916-38 | 1.12 | 1.05 | 1.20 | 1.13 | 1.09 | 1.16 | 1.03 | 0.91 | 1.16 | 1.04 | 0.99 | 1.10 |
| 1925-42 | 1.17 | 1.10 | 1.25 | 1.13 | 1.10 | 1.17 | 1.05 | 0.94 | 1.18 | 1.08 | 1.02 | 1.14 |
| 1940-46 | 1.15 | 1.08 | 1.22 | 1.11 | 1.08 | 1.14 | 1.01 | 0.91 | 1.13 | 1.08 | 1.03 | 1.14 |
| 1944-50 | 1.11 | 1.05 | 1.19 | 1.13 | 1.10 | 1.17 | 1.02 | 0.92 | 1.14 | 1.11 | 1.06 | 1.17 |
| 1948-54 | 1.13 | 1.06 | 1.20 | 1.15 | 1.12 | 1.18 | 1.04 | 0.94 | 1.15 | 1.15 | 1.10 | 1.20 |
| 1952-58 | 1.17 | 1.11 | 1.24 | 1.16 | 1.13 | 1.19 | 1.05 | 0.96 | 1.16 | 1.17 | 1.12 | 1.21 |
| 1956-62 | 1.12 | 1.06 | 1.18 | 1.13 | 1.11 | 1.16 | 1.09 | 1.00 | 1.19 | 1.17 | 1.13 | 1.21 |
| 1960-66 | 1.09 | 1.04 | 1.15 | 1.13 | 1.11 | 1.15 | 1.04 | 0.96 | 1.12 | 1.16 | 1.12 | 1.20 |
| 1964-70 | 1.07 | 1.02 | 1.12 | 1.07 | 1.05 | 1.09 | 1.06 | 0.99 | 1.14 | 1.11 | 1.08 | 1.14 |
| 1968-74 | 0.99 | 0.95 | 1.03 | 1.00 | 0.99 | 1.02 | 0.97 | 0.91 | 1.03 | 1.05 | 1.02 | 1.07 |
| 1972-78 | 0.92 | 0.88 | 0.95 | 0.94 | 0.93 | 0.96 | 0.94 | 0.89 | 0.99 | 0.99 | 0.96 | 1.01 |
| 1976-82 | 0.86 | 0.82 | 0.89 | 0.92 | 0.90 | 0.93 | 0.88 | 0.82 | 0.94 | 0.96 | 0.93 | 0.98 |
| 1980-86 | 0.84 | 0.79 | 0.88 | 0.90 | 0.88 | 0.92 | 0.88 | 0.82 | 0.95 | 0.92 | 0.89 | 0.94 |
| 1984-90 | 0.87 | 0.81 | 0.93 | 0.88 | 0.86 | 0.90 | 0.90 | 0.82 | 0.99 | 0.87 | 0.84 | 0.91 |
| 1988-94 | 0.82 | 0.76 | 0.90 | 0.86 | 0.83 | 0.89 | 0.85 | 0.75 | 0.98 | 0.84 | 0.79 | 0.88 |
| 1992-98 | 0.82 | 0.73 | 0.92 | 0.80 | 0.76 | 0.84 | 0.88 | 0.74 | 1.05 | 0.77 | 0.71 | 0.83 |
| 1996-02 | 0.70 | 0.57 | 0.85 | 0.81 | 0.74 | 0.88 | 0.66 | 0.48 | 0.91 | 0.74 | 0.65 | 0.84 |

Relative risks obtained from age-period-cohort intrinsic estimator models adjusted for driver sex, province and number of fatalities or serious injuries divided by the remaining persons involved in the same crash (0, 1, 2, 3 or more).

RD: Risk of death within 24 hours. RDH: Risk of death or hospitalisation.
